# Reduced proliferation in breast cancer cells contacting the neighboring adipocytes in human breast cancer tissues

**DOI:** 10.1186/s13058-015-0602-3

**Published:** 2015-07-02

**Authors:** Han Suk Ryu, Han-Byoel Lee, Wonshik Han, Dong-Young Noh, Hyeong-Gon Moon

**Affiliations:** Department of Pathology, Seoul National University College of Medicine, 28 Yongon-dong, Chongno-gu, Seoul 110-744 South Korea; Department of Surgery, Seoul National University College of Medicine, 28 Yongon-dong, Chongno-gu, Seoul 110-744 South Korea; Laboratory of Breast Cancer Biology, Cancer Research Institute, Seoul National University College of Medicine, 28 Yongon-dong, Chongno-gu, Seoul 110-744 South Korea

The process of tumor formation and progression is a result of the complex interaction between malignant epithelial cells and the various cell populations in the tumor microenvironment [1, 2]. Recently, the role of cancer-associated adipocytes in cancer progression has been an active area of research, and many studies have suggested a proinvasive and prometastatic effect of the cancer-associated adipocytes [3, 4]. The cancer-promoting effect of adipocytes seems to originate from their ability to induce inflammatory milieu and to provide an energy source for cancer cells [5, 6].

**Fig. 1:**
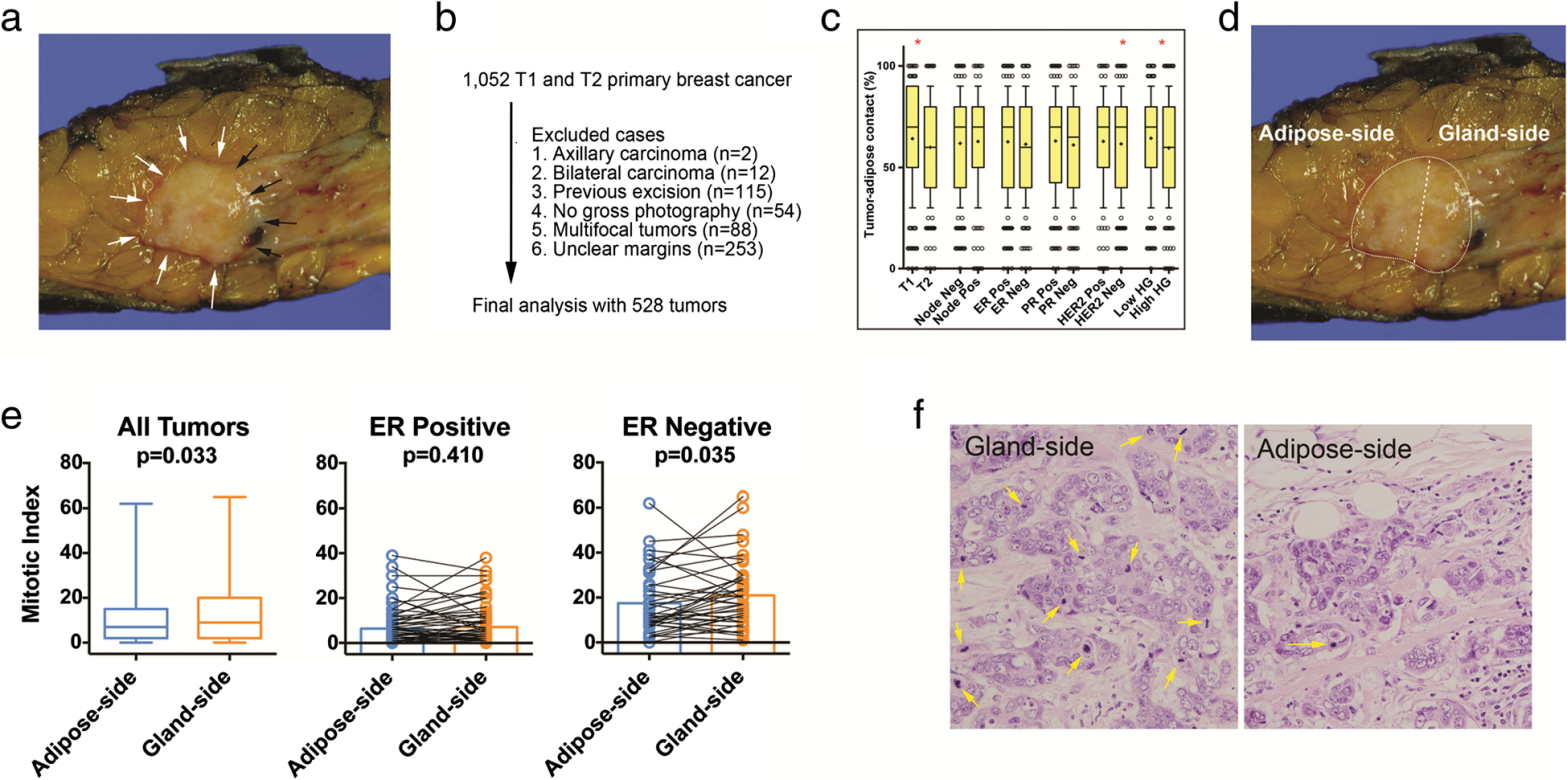
Adipose–tumor interaction and the histologic grades of tumor cells. **a** Representative gross photograph showing the tumor area in contact with surrounding adipose tissue (white arrows) and gland tissue (black arrows). **b** Design and exclusion criteria. **c** Association between various clinico-pathologic factors and the degree of tumor–adipose contact. *P < 0.05. ER, estrogen receptor; HER2, human epidermal growth factor 2; HG, histologic grade; PR, progesterone receptor. **d** In 107 patients whose tumor showed moderate tumor–adipose interaction, the degrees of mitotic index, nuclear pleomorphism, and tubule formation were measured in adipose-side tumors and gland-side tumors. **e** The gland-side tumor cells showed significantly higher mitotic index especially in ER-negative tumors, suggested by the changes in mitotic index within same tumors. *P* values derived from the comparison using the Wilcoxon signed-rank test. **f** Representative case of a tumor with significant changes in mitotic index according to the tumor cells’ location with regard to surrounding adipose tissues

Human breast tissue has a unique architecture of adipose tissue surrounding the mammary glandular tissues. Many human breast cancers face the adipose tissue during their growth, and therefore there is direct contact between cancer cells and adipocytes. We have reviewed representative photographs of the 1,052 macroscopic surgical specimens of breast cancer resected between January 2006 and July 2007. This study was approved by the institutional review board (1208-046-421). In 528 patients, we were able to measure the relative ratio of tumor–adipose contact among the total circumference (Fig. 1a for a representative case and Fig. 1b for exclusion criteria), and the association between various clinico-pathological factors and the degree of tumor–adipose contact was analyzed. Tumors with higher degree of direct tumor–adipose contact were more likely to be smaller tumors and low-grade tumors (Fig. 1c).

To overcome the issue of intertumoral heterogeneity, we semiquantitatively analyzed the histologic grade of cancer cells on the adipose side and on the gland side within the same tumor in 107 patients whose tumors had moderate tumor–adipose contact (between 0.4 and 0.6; Fig. 1d). We measured the degree of the tubule formation, nuclear pleomorphism, and mitotic count, which are the three components of the widely used Elston–Ellis modification of the Scarff–Bloom–Richardson histologic grading system for both adipose-side and gland-side tumor cells [7]. The tumor cells on the adipose side showed significantly lower mitotic index when compared with that of the tumor cells on the gland side. The significant association with mitotic index and the cancer cells’ distance to the adipose tissue was only seen in estrogen receptor-negative breast tumors (Fig. 1e, representative case shown in Fig. 1f). There was no significant difference between the adipose-side and gland-side cells in tubule formation and nuclear pleomorphism.

In conclusion, our analysis of human breast cancer samples showed that tumor cells residing close to the adipose tissue showed significantly lower mitotic count than cells distant from the adipose tissue. Our findings suggest that, contrary to the prevailing concept of the cancer-promoting role of cancer-associated adipocytes, the true interaction between cancer cells and the neighboring adipose tissue can be a complex one.
